# Phantom‐based gradient waveform measurements with compensated variable‐prephasing: Description and application to EPI at 7 T

**DOI:** 10.1002/mrm.30425

**Published:** 2025-01-20

**Authors:** Hannah Scholten, Tobias Wech, Istvan Homolya, Herbert Köstler

**Affiliations:** ^1^ Department of Diagnostic and Interventional Radiology University Hospital Würzburg Würzburg Germany; ^2^ Comprehensive Heart Failure Center University Hospital Würzburg Würzburg Germany; ^3^ Chair of Molecular and Cellular Imaging, Comprehensive Heart Failure Center University Hospital Würzburg Würzburg Germany

**Keywords:** EPI, gradient impulse response, gradient measurement, ultrahigh field, variable‐prephasing

## Abstract

**Purpose:**

Introducing compensated variable‐prephasing (CVP), a phantom‐based method for gradient waveform measurements. The technique is based on the variable‐prephasing (VP) method, but takes into account the effects of all gradients involved in the measurement.

**Methods:**

We conducted measurements of a trapezoidal test gradient and of an EPI readout gradient train with three approaches: VP, CVP, and fully compensated variable‐prephasing (FCVP). We compared them to one another and to predictions based on the gradient system transfer function. Furthermore, we used the measured and predicted EPI gradients for trajectory corrections in phantom images on a 7 T scanner.

**Results:**

The VP gradient measurements are confounded by lingering oscillations of the prephasing gradients, which are compensated in the CVP and FCVP measurements. FCVP is vulnerable to a sign asymmetry in the gradient chain. However, the trajectories determined by all three methods resulted in comparably high EPI image quality.

**Conclusion:**

We present a new approach allowing for phantom‐based gradient waveform measurements with high precision, which can be useful for trajectory corrections in non‐Cartesian or single‐shot imaging techniques. In our experimental setup, the proposed “compensated variable‐prephasing” method provided the most reliable gradient measurements of the different techniques we compared.

## 1 INTRODUCTION

The dynamically switching gradient fields of an MRI scanner can suffer from temporal errors because of hardware delays, eddy currents, coil vibrations, or other system imperfections. Uncorrected, erroneous gradient waveforms can cause image artifacts, especially in non‐Cartesian or single‐shot techniques. Therefore, methods to obtain precise knowledge of the actual gradient field evolution become increasingly important and popular.

Field cameras are one possibility to measure the true gradient waveforms in the bore of the scanner.^1^, ^2^ Another method using only standard scanner hardware is the thin‐slice method, where the gradient waveform is inferred from the change in the phase of an FID signal measured in a thin off‐center slice.^3^ However, this approach is limited by the gradient‐induced signal dephasing.^4^ Another gradient measurement principle is summarized under the term self‐encoding methods.^4^, ^5^, ^6^, ^7^, ^8^ They apply dephasing gradients with defined integral between a slice‐selective excitation and the FID readout during which the gradient of interest is applied. By repeating this with a sufficient number of different amplitudes of the self‐encoding gradient, the test gradient waveform can be inferred from the envelope of the magnitude signals. The drawback is the long acquisition time, because it requires a large number of repetitions. Recently, Harkins and Does^4^ published a hybrid method combining the thin‐slice method with the self‐encoding approach. By applying self‐encoding gradients of variable amplitudes in repeated acquisitions of the thin‐slice signal, their variable‐prephasing (VP) approach enables gradient waveform measurements in thicker slices with higher SNR than in the original thin‐slice method, while keeping the scan time relatively short. However, they neglect the effects of the “prephasing” gradients, such as lingering eddy currents or oscillations, which can lead to inaccurate results when they last long enough to superimpose the gradient waveform of interest.^9^

Here, we present compensated variable‐prephasing (CVP), an extension of the VP method^4^ for phantom‐based measurements of gradient waveforms. Our approach allows for the compensation of lingering repercussions of the prephasing gradients, which are neglected in the original method. Another method that we developed in a previous study^10^ and term fully compensated variable‐prephasing (FCVP) additionally compensates for concomitant field effects. We evaluate the differences between the three methods and compare their results to the predictions of a linear time‐invariant model of the gradient system,^11^ which is represented by the gradient system transfer function (GSTF).^12^, ^13^, ^14^ We also assess how the differences between the presented methods affect image reconstructions by conducting EPI experiments in a phantom and applying trajectory corrections based on these methods. Parts of this work have been presented at the Annual Meeting of the International Society for Magnetic Resonance in Medicine (ISMRM) 2023.^9^

## 2 THEORY

**FIGURE 1:**
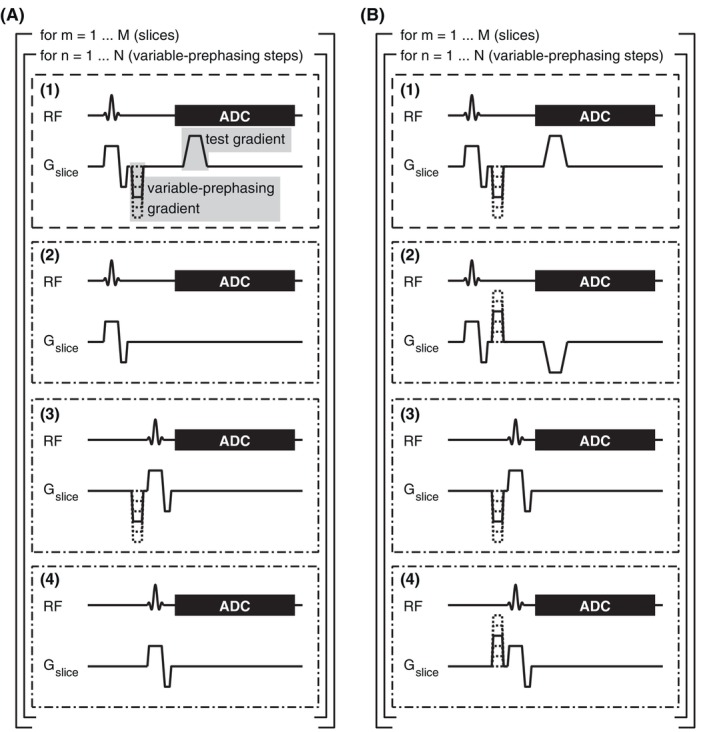
Sequence diagrams demonstrating the measurement schemes of our (A) compensated variable‐prephasing (CVP) and (B) fully compensated variable‐prephasing (FCVP) approaches. CVP accounts for lingering effects of the slice selection and the prephasing gradients. FCVP additionally compensates concomitant field effects.

Variable‐prephasing^4^ constitutes an extension of the thin‐slice method^3^, ^15^ and is briefly reviewed before introducing “compensated” and “fully compensated variable‐prephasing.” In the thin‐slice method, slice‐selective excitations are followed by FID readouts during which the gradient waveform of interest is played out. It is determined from the phase difference of the signals in the excited slices. Depending on the applied gradient moment and the slice thickness, complete signal dephasing can eventually occur, preventing a meaningful phase extraction from the signal. VP lifts this limitation by adding prephasing gradients of variable amplitude between the excitation and the test gradient. This creates a maximum in the acquired FID signal when the integral of the test gradient waveform cancels the negative integral of the prephasing gradient. Repeating the acquisition with different prephasing amplitudes results in maxima at different time points during the test gradient and allows compensating for its dephasing effect. The acquisition scheme is depicted in boxes (1) and (2) of Figure 1A. A reference acquisition without the prephasing and test gradients is used to account for effects from the slice selection gradient (box 2).

Addressing effects from the prephasing gradients in addition to effects from the slice selection is the main purpose of the proposed CVP. We, therefore, added the steps in boxes (3) and (4) of Figure 1A to the measurement scheme. In step (3), applying the prephasing gradient before the excitation allows to capture lingering effects from the prephasers at the time of the FID readout.^10^, ^16^ The effects of the slice selection gradient in step (3) are recorded in step (4). The timing of the prephasing gradients and the readout is identical in all four steps.

To transfer into FCVP, we modified steps (2) and (4) to allow removing concomitant field effects from the waveform, similar to the method published by Brodsky et al.^15^ Because concomitant gradient fields depend approximately quadratically on the gradient amplitude,^17^ they can be canceled out by subtracting the signals from acquisitions with inverted gradients. This is done for the test gradient and prephasing gradients in the measurement scheme shown in Figure 1B. In the following, we develop the mathematical description of FCVP and successively reduce it to obtain the formulas for CVP and VP.

First, we take the time derivative of the phase of the measured FID signal. This frequency evolution f(r,t) is described by the following equations in the four measurement steps of the nth prephasing step in Figure 1B:

(1) $$\begin{array}{rl} & \;f_{n,1}\left(r,t\right)=\frac{\gamma }{2\pi }\left[\sum_{j=1}^{2}p_{j}\left(r\right)\left(d_{j}\left(t\right)+d_{j,n}^{\mathrm{VP}}\left(t\right)\right)\right]+c\left(r,t\right)+c_{n}^{\mathrm{VP}}\left(r,t\right)+q^{I}\left(r,t\right)\\ \text{with} & \;p_{1}\left(r\right)=1,p_{2}\left(r\right)=r,d_{1}\left(t\right)=\Delta B_{0}\left(t\right),d_{2}\left(t\right)=G\left(t\right),d_{1,n}^{\mathrm{VP}}\left(t\right)=\Delta B_{0,n}^{\mathrm{VP}}\left(t\right),d_{2,n}^{\mathrm{VP}}\left(t\right)=G_{n}^{\mathrm{VP}}\left(t\right) \end{array},$$

(2) $$f_{n,2}\left(r,t\right)=\frac{\gamma }{2\pi }\left[\sum_{j=1}^{2}p_{j}\left(r\right)\left(-d_{j}\left(t\right)-d_{j,n}^{\mathrm{VP}}\left(t\right)\right)\right]+c\left(r,t\right)+c_{n}^{\mathrm{VP}}\left(r,t\right)+q^{I}\left(r,t\right),$$

(3) $$f_{n,3}\left(r,t\right)=\frac{\gamma }{2\pi }\left[\sum_{j=1}^{2}p_{j}\left(r\right)d_{j,n}^{\mathrm{VP}}\left(t\right)\right]+c_{n}^{\mathrm{VP}}\left(r,t\right)+q^{\mathrm{II}}\left(r,t\right),$$

(4) $$f_{n,4}\left(r,t\right)=\frac{\gamma }{2\pi }\left[\sum_{j=1}^{2}p_{j}\left(r\right)\left(-d_{j,n}^{\mathrm{VP}}\left(t\right)\right)\right]+c_{n}^{\mathrm{VP}}\left(r,t\right)+q^{\mathrm{II}}\left(r,t\right),$$

(r: the slice position, $p_{\left\{1,2\right\}}\left(r\right)$: spatial basis functions,^11^ $d_{\left\{1,2\right\}}\left(t\right)$ and $d_{\left\{1,2\right\},n}^{\mathrm{VP}}\left(t\right)$: corresponding field coefficients referring to the test gradient and the nth prephasing gradient, c(r,t) and $c_{n}^{\mathrm{VP}}\left(r,t\right)$: phase contributions from concomitant fields, $q^{I}\left(r,t\right)$ and $q^{\mathrm{II}}\left(r,t\right)$: background terms related to the slice selection.) By stacking the equations for the acquisitions from M slice positions, we obtain Eq. (5).

(5) $$\left[\begin{array}{c} \begin{array}{c} \begin{array}{c} \begin{array}{c} f_{1,1}\\ f_{2,1} \end{array}\\ \begin{array}{c} \vdots \\ f_{N,1} \end{array} \end{array}\\ \begin{array}{c} \begin{array}{c} f_{1,2}\\ f_{2,2} \end{array}\\ \begin{array}{c} \vdots \\ f_{N,2} \end{array} \end{array} \end{array}\\ \begin{array}{c} \begin{array}{c} \begin{array}{c} f_{1,3}\\ f_{2,3} \end{array}\\ \begin{array}{c} \vdots \\ f_{N,3} \end{array} \end{array}\\ \begin{array}{c} \begin{array}{c} f_{1,4}\\ f_{2,4} \end{array}\\ \begin{array}{c} \vdots \\ f_{N,4} \end{array} \end{array} \end{array} \end{array}\right]=\left[\begin{array}{ccccccccccccc} P & P & 0 & \cdots & 0 & I_{M} & 0 & \cdots & 0 & 0 & 0 & \cdots & 0\\ P & 0 & P & \cdots & 0 & 0 & I_{M} & \cdots & 0 & 0 & 0 & \cdots & 0\\ \vdots & \vdots & \vdots & \ddots & \vdots & \vdots & \vdots & \ddots & \vdots & \vdots & \vdots & \ddots & \vdots \\ P & 0 & 0 & \cdots & P & 0 & 0 & \cdots & I_{M} & 0 & 0 & \cdots & 0\\ -\;P & -P & 0 & \cdots & 0 & I_{M} & 0 & \cdots & 0 & 0 & 0 & \cdots & 0\\ -\;P & 0 & -P & \cdots & 0 & 0 & I_{M} & \cdots & 0 & 0 & 0 & \cdots & 0\\ \vdots & \vdots & \vdots & \ddots & \vdots & \vdots & \vdots & \ddots & \vdots & \vdots & \vdots & \ddots & \vdots \\ -\;P & 0 & 0 & \cdots & -P & 0 & 0 & \cdots & I_{M} & 0 & 0 & \cdots & 0\\ 0 & P & 0 & \cdots & 0 & 0 & 0 & \cdots & 0 & I_{M} & 0 & \cdots & 0\\ 0 & 0 & P & \cdots & 0 & 0 & 0 & \cdots & 0 & 0 & I_{M} & \cdots & 0\\ \vdots & \vdots & \vdots & \ddots & \vdots & \vdots & \vdots & \ddots & \vdots & \vdots & \vdots & \ddots & \vdots \\ 0 & 0 & 0 & \cdots & P & 0 & 0 & \cdots & 0 & 0 & 0 & \cdots & I_{M}\\ 0 & -P & 0 & \cdots & 0 & 0 & 0 & \cdots & 0 & I_{M} & 0 & \cdots & 0\\ 0 & 0 & -P & \cdots & 0 & 0 & 0 & \cdots & 0 & 0 & I_{M} & \cdots & 0\\ \vdots & \vdots & \vdots & \ddots & \vdots & \vdots & \vdots & \ddots & \vdots & \vdots & \vdots & \ddots & \vdots \\ 0 & 0 & 0 & \cdots & -P & 0 & 0 & \cdots & 0 & 0 & 0 & \cdots & I_{M} \end{array}\right]\left[\begin{array}{c} d\\ d_{1}^{\mathrm{VP}}\\ d_{2}^{\mathrm{VP}}\\ \vdots \\ d_{N}^{\mathrm{VP}}\\ c_{1}^{I}\\ c_{2}^{I}\\ \vdots \\ c_{N}^{I}\\ c_{1}^{\mathrm{II}}\\ c_{2}^{\mathrm{II}}\\ \vdots \\ c_{N}^{\mathrm{II}} \end{array}\right]$$

$$\text{with }\;\;f_{n,k}=\left[\begin{array}{c} f_{n,k}\left(r_{1},t\right)\\ f_{n,k}\left(r_{2},t\right)\\ \vdots \\ f_{n,k}\left(r_{m},t\right)\\ \vdots \\ f_{n,k}\left(r_{M},t\right) \end{array}\right],P=\left[\begin{array}{cc} 1 & r_{1}\\ 1 & r_{2}\\ \vdots & \vdots \\ 1 & r_{m}\\ \vdots & \vdots \\ 1 & r_{M} \end{array}\right],d=\left[\begin{array}{c} \Delta B_{0}\left(t\right)\\ G\left(t\right) \end{array}\right],d_{n}^{\mathrm{VP}}=\left[\begin{array}{c} \Delta B_{0,n}^{\mathrm{VP}}\left(t\right)\\ G_{n}^{\mathrm{VP}}\left(t\right) \end{array}\right],$$

(6) $$c_{n}^{I}=\left[\begin{array}{c} c\left(r_{1},t\right)+c_{n}^{\mathrm{VP}}\left(r_{1},t\right)+q^{I}\left(r_{1},t\right)\\ c\left(r_{2},t\right)+c_{n}^{\mathrm{VP}}\left(r_{2},t\right)+q^{I}\left(r_{2},t\right)\\ \vdots \\ c\left(r_{m},t\right)+c_{n}^{\mathrm{VP}}\left(r_{m},t\right)+q^{I}\left(r_{m},t\right)\\ \vdots \\ c\left(r_{M},t\right)+c_{n}^{\mathrm{VP}}\left(r_{M},t\right)+q^{I}\left(r_{M},t\right) \end{array}\right],\;c_{n}^{\mathrm{II}}=\left[\begin{array}{c} c_{n}^{\mathrm{VP}}\left(r_{1},t\right)+q^{\mathrm{II}}\left(r_{1},t\right)\\ c_{n}^{\mathrm{VP}}\left(r_{2},t\right)+q^{\mathrm{II}}\left(r_{2},t\right)\\ \vdots \\ c_{n}^{\mathrm{VP}}\left(r_{m},t\right)+q^{\mathrm{II}}\left(r_{m},t\right)\\ \vdots \\ c_{n}^{\mathrm{VP}}\left(r_{M},t\right)+q^{\mathrm{II}}\left(r_{M},t\right) \end{array}\right]$$

(n=1,…,N: index of the prephasing step, m=1,…,M: slice number, $I_{M}$: M×M identity matrix, k=1,2,3,4 iterates through the acquisition steps in Figure 1B).

For CVP (Figure 1A), Eq. (2) reduces to $f_{n,2}\left(r,t\right)=q^{I}\left(r,t\right)$ and Eq. (4) to $f_{n,4}\left(r,t\right)=q^{\mathrm{II}}\left(r,t\right)$. To obtain a well‐posed system of equations, we neglect the contributions from concomitant fields, that is, $c\left(r,t\right)=c_{n}^{\mathrm{VP}}\left(r,t\right)=0$, arriving at Eq. (7). Note that, in reality, this assumption might not always be valid.

(7) $$\left[\begin{array}{c} \begin{array}{c} \begin{array}{c} \begin{array}{c} f_{1,1}\\ f_{2,1} \end{array}\\ \begin{array}{c} \vdots \\ f_{N,1} \end{array} \end{array}\\ \begin{array}{c} \begin{array}{c} f_{1,2}\\ f_{2,2} \end{array}\\ \begin{array}{c} \vdots \\ f_{N,2} \end{array} \end{array} \end{array}\\ \begin{array}{c} \begin{array}{c} \begin{array}{c} f_{1,3}\\ f_{2,3} \end{array}\\ \begin{array}{c} \vdots \\ f_{N,3} \end{array} \end{array}\\ \begin{array}{c} \begin{array}{c} f_{1,4}\\ f_{2,4} \end{array}\\ \begin{array}{c} \vdots \\ f_{N,4} \end{array} \end{array} \end{array} \end{array}\right]=\left[\begin{array}{ccccccc} P & P & 0 & \cdots & 0 & I_{M} & 0\\ P & 0 & P & \cdots & 0 & I_{M} & 0\\ \vdots & \vdots & \vdots & \ddots & \vdots & \vdots & \vdots \\ P & 0 & 0 & \cdots & P & I_{M} & 0\\ 0 & 0 & 0 & \cdots & 0 & I_{M} & 0\\ 0 & 0 & 0 & \cdots & 0 & I_{M} & 0\\ \vdots & \vdots & \vdots & \ddots & \vdots & \vdots & \vdots \\ 0 & 0 & 0 & \cdots & 0 & I_{M} & 0\\ 0 & P & 0 & \cdots & 0 & 0 & I_{M}\\ 0 & 0 & P & \cdots & 0 & 0 & I_{M}\\ \vdots & \vdots & \vdots & \ddots & \vdots & \vdots & \vdots \\ 0 & 0 & 0 & \cdots & P & 0 & I_{M}\\ 0 & 0 & 0 & \cdots & 0 & 0 & I_{M}\\ 0 & 0 & 0 & \cdots & 0 & 0 & I_{M}\\ \vdots & \vdots & \vdots & \ddots & \vdots & \vdots & \vdots \\ 0 & 0 & 0 & \cdots & 0 & 0 & I_{M} \end{array}\right]\left[\begin{array}{c} d\\ d_{1}^{\mathrm{VP}}\\ d_{2}^{\mathrm{VP}}\\ \vdots \\ d_{N}^{\mathrm{VP}}\\ q^{I}\\ q^{\mathrm{II}} \end{array}\right]$$

(8) $$\begin{array}{cc} \text{with } & f_{n,k},\;P,\;d,\;d_{n}^{\mathrm{VP}}\text{ as in Eq. (6), and }q^{I}=\\ & \left[\begin{array}{c} q^{I}\left(r_{1},t\right)\\ q^{I}\left(r_{2},t\right)\\ \vdots \\ q^{I}\left(r_{m},t\right)\\ \vdots \\ q^{I}\left(r_{M},t\right) \end{array}\right],\;q^{\mathrm{II}}=\left[\begin{array}{c} q^{\mathrm{II}}\left(r_{1},t\right)\\ q^{\mathrm{II}}\left(r_{2},t\right)\\ \vdots \\ q^{\mathrm{II}}\left(r_{m},t\right)\\ \vdots \\ q^{\mathrm{II}}\left(r_{M},t\right) \end{array}\right] \end{array}$$

The VP solution is derived from measurement steps (1) and (2) in Figure 1A by solving the following matrix equation:

(9) $$\left[\begin{array}{c} \begin{array}{c} \begin{array}{c} f_{1,1}\\ f_{2,1} \end{array}\\ \begin{array}{c} \vdots \\ f_{N,1} \end{array} \end{array}\\ \begin{array}{c} \begin{array}{c} f_{1,2}\\ f_{2,2} \end{array}\\ \begin{array}{c} \vdots \\ f_{N,2} \end{array} \end{array} \end{array}\right]=\left[\begin{array}{cc} P & I_{M}\\ P & I_{M}\\ \vdots & \vdots \\ P & I_{M}\\ 0 & I_{M}\\ 0 & I_{M}\\ \vdots & \vdots \\ 0 & I_{M} \end{array}\right]\left[\begin{array}{c} d\\ q^{I} \end{array}\right],$$

(10) $$\text{with }f_{n,k},\;P,\;d,\;\text{ and }q^{I}\;\text{as in Eq. (8). }$$

We solved Eqs. (5), (7), and (9) for the weighted least squares solution d(t) for each time point, similar to Harkins and Does.^4^ Rewriting each matrix equation as f=Ab, the weighted least squares solution is given by^18^

(11) $$\hat{b}={\left(A^{T}\sum^{-1}A\right)}^{-1}\;A^{T}\sum^{-1}f.$$

$\sum^{-1}$ denotes the inverse covariance matrix of f. The entries of f are time derivatives of signal phases. The variance of the phase of an MR signal can be approximated by

(12) $$\sigma_{\phi }^{2}\approx \frac{\sigma^{2}}{{\left|S\right|}^{2}},$$

where $\sigma^{2}$ denotes the variance of the Gaussian noise of the real or imaginary part of the measured MR signal, assuming they are equal, and ∣S∣ denotes the signal magnitude.^19^ Therefore, the entries of the diagonal covariance matrix $\sum^{-1}$ are proportional to the respective squared signal magnitudes, and the proportionality constants cancel in Eq. (11). The weights for the weighted least squares solution are, therefore, given by the measured signal magnitudes.

## 3 METHODS

### 3.1 Gradient waveform measurements

First, we measured the waveform of a trapezoidal test gradient with VP, CVP, and FCVP with the sequence parameters specified in Table 1. The acquisitions in each slice were preceded by 10 dummy excitations to reach a steady‐state magnetization. The VP solution of the gradient waveform was obtained only evaluating the readouts from steps (1) and (2) in Figure 1A according to Eq. (9). To increase robustness against noise, the measured frequency evolutions f(t) were treated with a moving median filter of length 3 before solving the matrixequations.

**TABLE 1:** Hardware and sequence parameters.

Hardware		
Main field strength	7 T	
Scanner model	MAGNETOM Terra (Siemens Healthineers)	
Receive coil	32‐channel head coil (Nova Medical)	
Max. gradient amplitude	80 mT/m	
Max. slew rate	200 T/m/s	
Gradient coil model	XR 80/200 (SC72CD coil)	
Spherical phantom diameter	165 mm	
Phantom filling	Polydimethylsiloxane oil	

Gradient waveform measurements		
	Trapezoid	EPI readout gradient
Max. amplitude	42 mT/m	35 mT/m
Ramp time	220 μs	
Flat top time	550 μs	
TR	500 ms	
No. slices	9	
Slice positions	0, ±4.0, ±8.0, ±12.5, and ±16.5 mm	
Slice thickness	2 mm	
Flip angle	90°	
No. prephasing steps	10	
Readout length	10.2 ms	100 ms
Dwell time	2.5 μs	5 μs

EPI image acquisition		
TR	100 ms	
TE	33 ms	
FOV readout direction	221 mm	
FOV phase encoding direction	80%	
Flip angle	90°	
Slice position	Isocenter	
Slice thickness	1.3 mm	
Matrix size	340 × 136	
Resolution	1.3 mm isotropic	
Partial Fourier factor	6/8	
Echo spacing	0.79 ms	
Receiver bandwidth	1470 Hz/pixel	
Dwell time	2 μs	
Ramp sampling	Yes	

*Note*: The built‐in eddy current compensation was active for all measurements.

Abbreviation: max., maximum.

Second, we measured the readout gradient train of an EPI readout. The parameters are given in Table 1.

We compared the measured gradient waveforms from VP, CVP, and FCVP to one another and to a prediction based on the GSTF.^11^ The Fourier transform of the nominal gradient waveform was multiplied by the GSTF of the respective axis, and the result transformed back to the time domain. The nominal waveform only consisted of the gradient of interest, without the prephasing or slice selection gradients. Dwell time differences were accounted for by adding a delay correction to the GSTF prediction (−1 μs for the trapezoid and − 6.3 μs for the EPI readout gradient). This counterbalances timing errors arising from interpolations between different time or frequency grids during the calculation^20^ (time resolution of nominal waveforms: 1 μs; GSTF: 9.8 μs; trapezoid measurement: 2.5 μs; EPI readout gradient measurement: 5 μs).

The GSTF was determined in a previous study.^10^ Triangular probing gradients were measured with the thin‐slice method with the same phantom and coil listed in Table 1 (with a dwell time of 9.8 μs). The measurement scheme was similar to FCVP without prephasing gradients, that is, steps (1) and (2) in Figure 1B. These triangle measurements were combined in a linear system of equations, from which the GSTF was calculated by a regularized matrix inversion^21^ and Fourier transform.

### 3.2 Image acquisition and reconstruction

We acquired a single‐shot EPI image with the parameters in Table 1 (transversal slice, phase encoding in anterior–posterior direction) and performed five reconstructions.

(1) For the first reconstruction, a constant phase shift, determined from the three reference echoes acquired before the EPI readout, was applied to the raw data of every second echo,^22^ and the nominal trajectory was used for image reconstruction.

(2) In the second reconstruction, the trajectory was calculated by integrating the GSTF‐predicted waveforms of the imaging gradients, calculated as described above.

(3, 4, 5) In the third, fourth, and fifth reconstruction, the readout gradient trains measured with VP, CVP, and FCVP and the nominal phase encoding gradients were used for the trajectory calculation.

We used the non‐uniform FFT toolbox^23^ from the Michigan Image Reconstruction Toolbox^24^ for image reconstruction.

In all but the first reconstruction, small additional delay corrections compensating dwell time differences were required to minimize ghosting artifacts. The corresponding gradient waveforms were, therefore, delayed before integration to obtain the k‐space trajectories. To optimize the delays, the image intensities outside a circular mask covering the phantom were summed up for different delays. The minimum of this sum was found by fitting a polynomial of fourth degree to the results and determining the roots of the first derivative. The corresponding delay was regarded as “optimal.”

The ghosting in the reconstructions was quantified by the relative ghost intensity Γ, which we calculated as the ratio of the maxima of the signal intensities in two regions of interest (ROIs): One in a region with well visible artifacts, but outside the imaged object (ROI 1), and the other one shifted by half the FOV in the phase encoding direction (ROI 2).

## 4 RESULTS

### 4.1 Gradient waveforms

**FIGURE 2:**
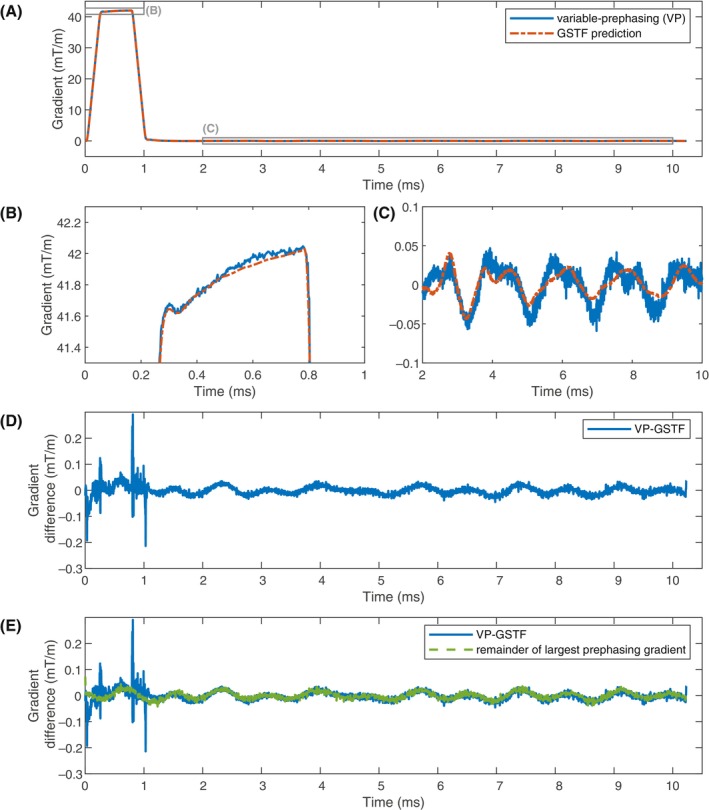
Comparison of the measurement of the trapezoidal test gradient with variable‐prephasing (VP) and the gradient system transfer function (GSTF) prediction. (A–C) Measured and predicted gradient waveforms. (D) Difference between VP‐measurement and GSTF prediction. The zoom in (C) and the difference in (D) reveal considerable discrepancies between the two gradient time courses in the lingering field oscillations after the test gradient is turned off. (E) Difference curve from (D) overlaid by the measured lingering oscillations of the largest prephasing gradient. For most of the measured time window, the two curves match well.

Figure 2 displays the VP measurement of the trapezoidal test gradient on the x‐axis and the corresponding GSTF prediction. There is a slight deviation between the two curves at approximately 0.6 ms (Figure 2B). In the lingering field oscillations after the gradient is turned off (Figure 2C), the VP‐measured gradient clearly differs from the GSTF prediction. Figure 2D shows the difference between measurement and prediction, which exhibits an oscillatory pattern. In Figure 2E, the difference is overlaid with the measurement of the largest prephasing gradient, extracted from the CVP measurement. Both curves match well, except for the time points coinciding with the ramps of the trapezoid.

**FIGURE 3:**
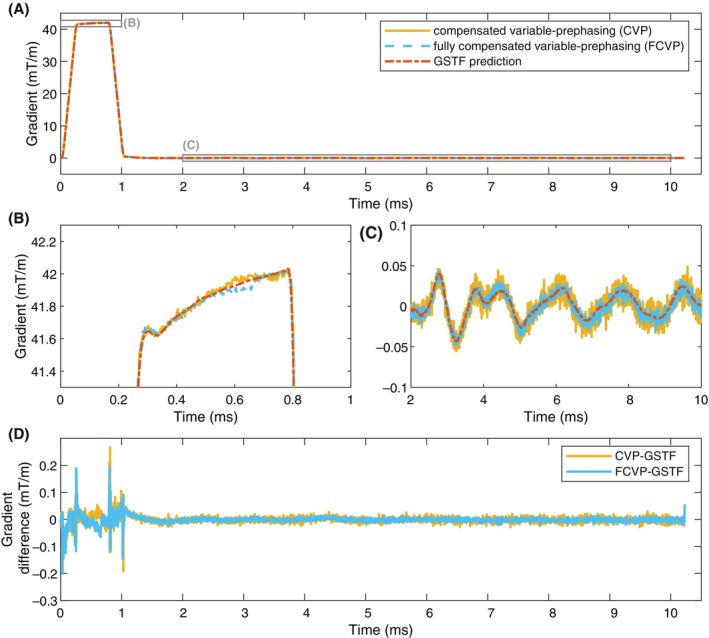
Comparison of the measurement of the trapezoidal test gradient with compensated variable‐prephasing (CVP), fully compensated variable‐prephasing (FCVP), and the gradient system transfer function (GSTF) prediction. (A–C) Measured and predicted gradient waveforms. (D) Differences between the measurements and the GSTF prediction. The predicted lingering field oscillations of the test gradient are replicated almost identically by the two measurement methods.

Figure 3 compares the CVP and FCVP measurements of the trapezoidal test gradient to the GSTF prediction. The CVP and FCVP measurements also slightly deviate from the GSTF prediction at around 0.6 ms (Figure 3B). In contrast to VP, both CVP and FCVP replicate the predicted lingering field oscillations shown in Figure 3C almost identically. This is confirmed by the difference curves (Figure 3D). The switching points of the gradient waveform again present as spikes in the difference.

**FIGURE 4:**
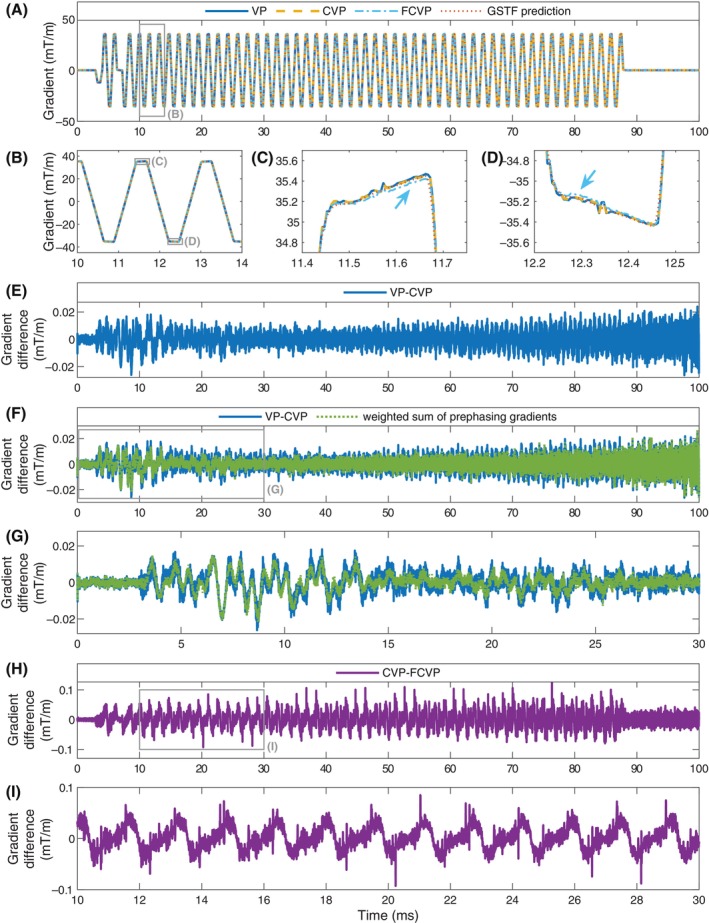
(A–D) EPI readout gradient waveform on the x‐axis of the gradient system, measured with variable‐prephasing (VP), compensated variable‐prephasing (CVP), and fully compensated variable‐prephasing (FCVP), and predicted by the gradient system transfer function (GSTF). The FCVP measurement differs visibly from the other three. (E–G) Difference between the VP and CVP measurement. In (F,G), it is overlaid with a weighted sum of the lingering oscillations from the prephasing gradients, which agrees well with the difference curve. (H,I) Difference between the CVP and FCVP measurement. Its repetitive structure resembles a sawtooth wave.

Figure 4A–D depict the different measurements and GSTF prediction of the EPI readout gradient on the x‐axis. The CVP and VP waveform agree well with each other and with the GSTF‐predicted waveform (Figure 4C,D). The FCVP waveform deviates visibly from these three for parts of the plateau phases of the gradient (blue arrows). The difference between the VP and CVP measurement (Figure 4E) exhibits an oscillatory pattern, similar to Figure 2D. In Figure 4F,G, this difference is overlaid by a weighted sum of the lingering oscillations from the prephasing gradients, extracted from the CVP measurement. The weights for each time point mimic how the different prephasing steps contribute to the weighted least squares solution of Eq. (9), that is, they are proportional to the quadratic signal magnitude of the respective prephasing step measured in box (1) in Figure 1A (averaged over the 9 slices). The two curves match well (Figure 4G). Figure 4H,I show the difference between the CVP and FCVP measurement of the EPI gradient. Its structure looks similar to a sawtooth wave. The observations made inFigure 4C,D, therefore, extend over the whole gradient train.

### 4.2 EPI images

**FIGURE 5:**
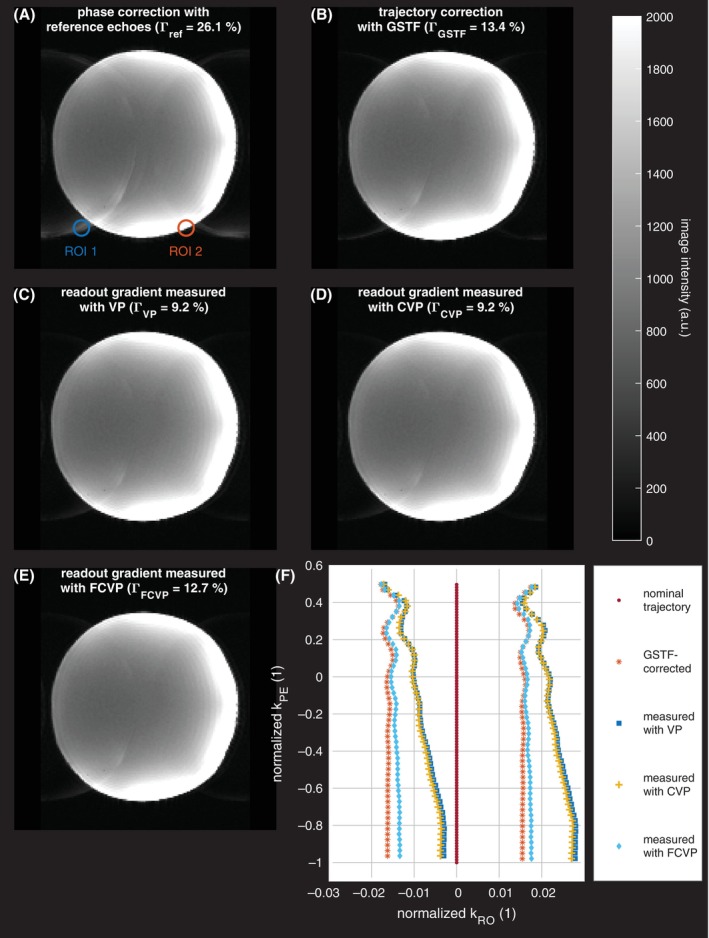
Transverse EPI images, reconstructed with the (A) navigator‐based, (B) gradient system transfer function (GSTF)‐based, (C) variable‐prephasing (VP) measurement‐based, (D) compensated variable‐prephasing (CVP) measurement‐based, and (E) fully compensated variable‐prephasing (FCVP) measurement‐based correction. Nyquist ghosts are most dominant in (A) and least visible in (C–E). The regions of interest (ROIs) in (A) were used for ghost quantification by means of the relative ghost intensity Γ. (F) k‐Space positions at the central points of each readout. k_RO_, k‐space position in readout direction; k_PE_, k‐space position in phase encoding direction. In the GSTF‐based and measurement‐based trajectories, the shift between odd and even echoes clearly oscillates at the beginning of the EPI echo train.

Figure 5A–E depict the transverse EPI images. Figure 5A shows the image resulting from the ghost correction method based on three reference echoes. Nyquist ghosts are clearly visible. The image in Figure 5B is reconstructed with the GSTF‐based reconstruction, where ghosting artifacts are greatly reduced. An additional delay correction of −0.99 μs was applied as described above. Without this delay, the ghosts had almost the same intensity as the actual phantom image. Figure 5C–E present the reconstructions with the VP‐, CVP‐, and FCVP‐measurements of the readout gradient, respectively, in which the Nyquist ghosts are even further reduced. The additional delay correction was 7.28 μs in the VP‐ and CVP‐based reconstructions and 7.27 μs for FCVP. Figure 5F displays the k‐space positions at the center point of each readout, that is, after half the readout duration, for the different reconstructions. The navigator‐based reconstruction assumes that $k_{\mathrm{RO}}=0$ at the center of each readout (red dots). In the GSTF‐based and measurement‐based reconstructions, we adjust the k‐space trajectory as described above. In all these cases, the centers of the odd and even readouts are shifted against each other, and the shift oscillates in the upper part of k‐space (normalized k_PE_ ≥ −0.2), which is sampled first after the excitation. For VP and CVP, there is an additional drift toward positive k_RO_‐values.

We determined a relative ghost intensity of $\Gamma_{\mathrm{ref}}=26.1\%$ for the ghost correction using reference echoes. In the GSTF‐based reconstruction, this value is approximately halved to $\Gamma_{\text{GSTF}}=13.0\%$ (without the additional delay correction, $\Gamma_{\text{GSTF}}=98.4\%$). Both the reconstructions with the VP‐ and CVP‐measured readout gradient exhibit the lowest relative ghost intensity of $\Gamma_{\mathrm{VP}}=\Gamma_{\mathrm{CVP}}=9.2\%$. With the FCVP measurement, the value is slightly higher ($\Gamma_{\text{FCVP}}=12.3\%$).

## 5 DISCUSSION AND CONCLUSIONS

We presented CVP, a new phantom‐based approach to determine the temporal evolution of magnetic gradient fields in MRI, which is based on the VP method.^4^ FCVP was introduced in a previous study^10^ and has now been examined more closely. In both CVP and FCVP, we compensate lingering effects of the prephasing gradients, which are neglected in VP. Applying all three methods to an EPI trajectory correction, we demonstrated that measuring the actual readout gradient yields superior suppression of ghosting artifacts compared to a navigator‐based phase correction or a GSTF‐based trajectory correction. However, the differences we detected between the three gradient waveform measurements did not translate to visible differences in the EPI images for our 7 T setup.

When comparing the VP measurement of a trapezoidal test gradient to the GSTF prediction, we noticed substantial differences in the lingering field oscillations after the gradient was switched off. They originated almost exclusively from the largest prephasing gradient, which can be explained by how Eq. (9) is solved. To find the weighted least squares solution to the matrix equation, the phase signals from the different prephasing gradients are weighted by their squared signal magnitude for each time point (Eqs. [11] and [12]). Because the largest prephasing gradient rephases the signal at the end of the test gradient, the signal magnitude is largest after the test gradient has been switched off. Therefore, the VP measurement superimposes the lingering oscillations of this prephasing gradient with those of the trapezoid itself.

In CVP and FCVP, the lingering oscillations of each prephasing gradient can be distinguished from the signal of the test gradient, so both measurements agree well with the GSTF prediction in the time window after the test gradient. Only when the gradient is active, we still see significant deviations between the measurements and the prediction. We attribute them to nonlinearities in the gradient amplifier behavior,^25^, ^26^, ^27^, ^28^ which can impact our observations in two ways.

(1) The nonlinearities are not replicated by the GSTF, causing inaccuracies in the gradient prediction.

(2) In both FCVP and the GSTF measurement, we use reference measurements in which the gradient of interest is inverted, to compensate for concomitant field effects. This compensation only works accurately when the actual output gradient with inverted sign is the exact negative of the non‐inverted output gradient. Nonlinearities in the gradient amplifiers can violate this assumption.

Because the feedback loop in the gradient amplifiers will always introduce certain nonlinearities into the gradient signal chain, we conclude that the CVP method yields the most accurate phantom‐based measurement of gradient waveforms at high and ultrahigh field. Concomitant fields of the lowest order are inversely proportional to $B_{0},$ ^17^ and can, therefore, be expected to be negligible above 1 T. At low (e.g., 0.55 T) or ultralow field, however, FCVP might be superior, because concomitant field contributions might supersede the nonlinearity effects. Further research is required to separate both effects. Looking at measurements of the gradient amplifiers' output currents could be a promising approach.^25^, ^26^, ^27^

Our measurements of an EPI readout gradient demonstrate that the uncompensated lingering oscillations of the prephasing gradients in the VP method can also alter the measurement result during active gradient switching. The systematic differences between the CVP and FCVP measurements of the EPI gradient confirm that the symmetry of positive and negative gradients, as assumed in FCVP, is violated. Uncompensated concomitant fields in CVP would cause the deviation to be in the same direction for positive and negative lobes of the EPI gradient, because concomitant fields of the lowest order depend quadratically on the gradient amplitude.^17^ However, the sign of the difference changes from positive to negative gradient lobes.

After demonstrating the high level of detail with which the presented measurement methods capture gradient evolutions, we chose an EPI application to evaluate the relevance of their precision for imaging. In EPI, gradient inaccuracies cause easily identifiable Nyquist ghosting (or N/2 ghosting).^29^ The current standard correction method for N/2 ghosts on most clinical MRI scanners uses two or three reference echoes to determine the shift between odd and even echoes.^22^ This shift is assumed constant for all phase encoding lines, which is reasonably fulfilled on 1.5 or 3 T magnets. However, it has been shown before that on human size 7 T systems, the shift between odd and even echoes varies substantially throughout the EPI readout,^30^, ^31^ leaving residual N/2 ghosts. Figure 5A confirms this, and Figure 5F demonstrates how the shift varies from line to line. For the GSTF predicted trajectory and the FCVP measurement, we observe a damped oscillation, as described before.^30^, ^31^ This oscillation occurs because the main frequency of the readout gradient is close to a mechanical resonance on the same gradient axis (here the x‐axis). The vibrations of the gradient coils are stronger at 7 T than at lower field strengths^16^, ^28^ or other systems (e.g., small‐animal scanners).^20^, ^32^ Therefore, precise knowledge of the actual k‐space trajectory can help remove ghosting from EPI on human size 7 T scanners.

In the VP and CVP measurements, the centrally sampled k‐space positions additionally drift in positive $k_{\mathrm{RO}}$ direction. A sheared k‐space trajectory principally results in a shear of the imaged object.^33^ In our experiments, this effect is negligibly small. Our quantitative evaluation revealed that the VP‐ and CVP‐measurements of the readout gradient work best for ghost suppression (relative ghost intensity, 9.2%). The reconstruction with the FCVP‐measurement exhibits a slightly higher relative ghost intensity of 12.3%, similar to the GSTF‐based reconstruction (13.0%). This correlates with the amount of drifting observed in the respective central readout points. Drifting could be related to the nonlinearities during gradient ramping. However, a detailed analysis of this matter is beyond the scope of this study. In summary, Figure 5 shows that gradient measurements with VP and CVP are equivalent concerning their application in EPI image reconstruction at our 7 T scanner. Using a GSTF‐predicted trajectory for image reconstruction yields slightly inferior ghost suppression, but is still considerably better than the navigator‐based method. The GSTF‐based ghost suppression could potentially be improved by including more different waveforms in the GSTF measurement.^34^ Especially adding trapezoids might reduce the differences between the predicted and actual EPI readout gradient caused by nonlinearities in the gradient chain. The quantitative results emphasize that the CVP method's relative insensitivity to gradient amplifier nonlinearities directly translates into its potential for artifact reduction.

Although we only analyzed first‐order gradient self‐terms, the proposed CVP method—as well as FCVP and VP—can be extended to determine higher‐order terms of the gradient field dynamics by adding phase encoding gradients to the sequence, similar to the approach for higher‐order GSTF measurements published by Rahmer et al.^35^ In Eqs. ((1), (2), (3), (4)), the sum over the spatial basis functions $p_{j}\left(r\right)$ simply has to be extended to higher indices, and the submatrix P has to be complemented by the appropriate basis functions.^11^

The B_0_ cross terms of the GSTF and gradient measurements were not corrected in this study because we observed considerable B_0_ cross term drifting on our scanner, prohibiting reliable and reproducible measurements of dynamic B_0_ shifting.

To avoid signal disturbance from B_0_ drift, all four steps in Figure 1 are executed immediately after each other for each slice and prephasing gradient. We, therefore, dedicate an equal amount of scan time to each step without considering if this is optimal with respect to SNR efficiency.^4^

Another limitation of this study is that both the GSTF‐based and the measurement‐based correction methods require additional delay corrections. A similar problem has been described for concurrent field monitoring using field probes.^36^

In conclusion, both CVP and FCVP expressed differences in the measured gradient waveforms compared to VP and the GSTF predictions. Because the FCVP approach is potentially compromised by a sign asymmetry in the gradient signal chain, we consider CVP the most reliable technique for our experimental setup. It enables a phantom‐based determination of the gradient field evolution with high precision, which can be useful for trajectory corrections in non‐Cartesian or single‐shot imaging. We demonstrated its application in single‐shot EPI imaging, where it proved to yield superior ghost suppression compared to the navigator‐based correction approach, a GSTF‐based trajectory correction, or correcting the trajectory with the FCVP measurement of the readout gradient.

## CONFLICT OF INTEREST STATEMENT

The University Hospital Würzburg has a research collaboration agreement with Siemens Healthineers AG.

## Data Availability

The measurement data and MATLAB code used in this work are publicly available at https://zenodo.org/doi/10.5281/zenodo.13742003.
